# The Effect of Sugammadex on Prothrombin and Activated Partial Thromboplastin Time

**DOI:** 10.7759/cureus.14521

**Published:** 2021-04-16

**Authors:** Evangelia Samara, Konstantinos Stamatiou, Marina Balanika, Petros Tzimas

**Affiliations:** 1 Department of Anaesthesiology, Onassis Cardiac Surgery Center, Athens, GRC; 2 Department of Urology, Tzaneio General Hospital of Piraeus, Piraeus, GRC; 3 Department of Anesthesiology and Postoperative Intensive Care, School of Health Sciences, University of Ioannina, Ioannina, GRC

**Keywords:** sugammadex, coagulation, bleeding, hemostasis, prothrombin time (pt), activated partial thromboplastin time (aptt)

## Abstract

Sugammadex is routinely used as an effective neuromuscular blockade reversal agent. Several studies have indicated that it may prolong the prothrombin time (PT) and the activated partial thromboplastin time (aPTT). This review gathers the relevant *in vivo* studies to accumulate knowledge on the subject. Nine studies were included. According to the results, sugammadex seems to lead to a transient increase in aPTT and PT values, compared to standard care. However, the clinical impact seems to be trivial. Nevertheless, the trials’ findings reveal great heterogeneity, preventing a meta-analysis. Therefore, more well-designed studies are needed to lead to prudent conclusions.

## Introduction and background

Sugammadex (Bridion®; Merck & Company, Inc., Whitehouse Station, NJ) is a modified γ-cyclodextrin that is routinely used in Europe as an effective reversal agent for rocuronium- and vecuronium-induced neuromuscular blockade since 2008. In December 2015, sugammadex was also approved by the Food and Drug Administration (FDA) for use in the United States after it was initially rejected over concerns regarding anaphylaxis, as well as the effects of sugammadex on coagulation [1].

Sugammadex binds and shifts the concentration gradient of rocuronium away from the neuromuscular junction [2]. Its efficacy and safety have been well-established, and it is associated with a lower risk of adverse events compared with neostigmine [3-4]. 

Although the most common adverse reactions reported are vomiting, dry mouth, tachycardia, hypotension [5], as well as anaphylactic reactions [6-7], several studies have indicated it may interfere with hemostasis by prolonging the prothrombin time (PT) and the activated partial thromboplastin time (aPTT) [8-9].

It is also recommended that “coagulation parameters should be carefully monitored in patients with known coagulopathies, being treated with therapeutic anticoagulation, receiving thromboprophylaxis drugs other than heparin and low molecular weight heparin, or receiving thromboprophylaxis drugs and who then receive a dose of 16 mg/kg sugammadex” [10].

In this review, we aimed to delineate the knowledge of the effect of sugammadex on PT and aPTT *in vivo*.

## Review

A search of PubMed, the online bibliographic database of the US National Library of Medicine was performed, using the following terms: sugammadex; coagulation; bleeding; hemostasis; prothrombin time; activated partial thromboplastin time. 

Inclusion criteria for this review were: (i) clinical studies evaluating sugammadex’s effect on hemostasis, (ii) in the English language, and (iii) conducted *in vivo*. 

Exclusion criteria were: (i) case reports and reviews, (ii) non-English language, (iii) studies *in vitro*, and (iv) studies evaluating hemostasis, not measuring PT and/ or aPTT.

Two investigators performed the search and selection to ensure that only relevant articles were included. Duplicate articles and articles not meeting the inclusion criteria were excluded from further analysis.

**Figure 1:**
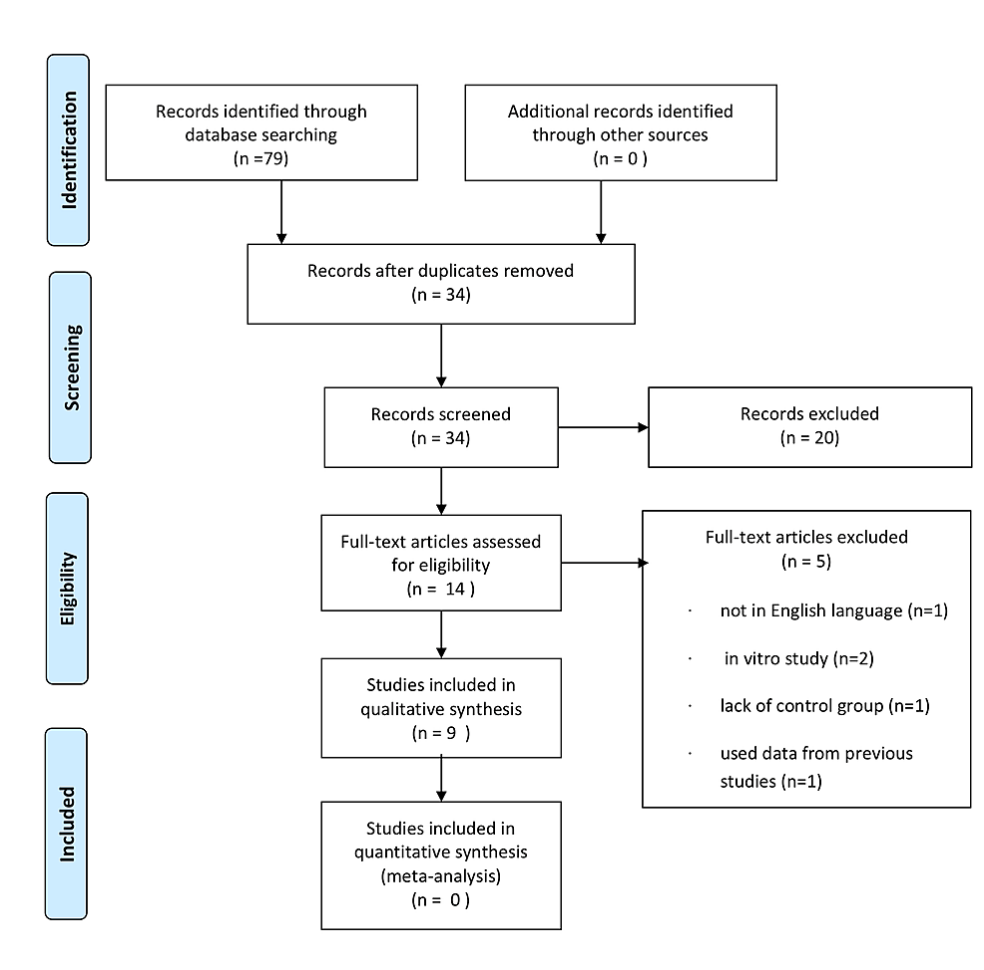
Preferred Reporting Items for Systematic Reviews and Meta-Analyses (PRISMA)-based flow diagram displaying the study selection

The two investigators excluded without disagreement 65 out of the 79 articles found in PubMed as duplicates or irrelevant and proceeded to the full-text reading of the remaining 14 articles. One study was excluded for not being written in the English language [11], three for having been conducted in an *in vitro* environment [12-14], and one for using data from previous studies to predict the outcome [15]. Nine studies were finally selected to assess the effect of sugammadex in aPTT and PT. The Preferred Reporting Items for Systematic Reviews and Meta-Analyses (PRISMA) flow diagram describing the steps of study selection can be found in Figure 1 [16].

In 2014, de Kam et al. measured PT and aPTT value changes in eight healthy volunteers after sugammadex 4 mg/kg or 16 mg/kg and placebo administration in a randomized crossover three-period study [8]. The endpoint was the area under the curve (AUC) 2 to 60 min after the dosing. According to the results, administration of sugammadex 4 mg/kg and 16 mg/kg led to a geometric mean ratio (sugammadex versus placebo) for AUC 2 to 60 min was 1.085 (95% CI 0.888 - 1.325) and 1.019 (0.868 - 1.195), respectively, for aPTT and 1.047 (0.904 - 1.213) and 1.096 (0.953 - 1.261), respectively, for PT (international normalized ratio (INR)). The mean value of aPTT and PT(INR) augmented up to 22% after sugammadex 16 mg/kg administration compared to placebo administration. All the above-mentioned differences occurred within 30 minutes after administration.

One year earlier, de Kam et al. evaluated the possibility of interaction between sugammadex and aspirin in platelet aggregation [9]. The researchers conducted a randomized, double-blind, placebo-controlled crossover study in 26 healthy male volunteers. The subjects received either intravenous placebo, sugammadex 4 mg/kg, or placebo/sugammadex with aspirin 75 mg, taken orally once a day. The primary endpoint was the interaction of sugammadex with aspirin in platelet aggregation. The effect in aPTT and bleeding time was also evaluated. Platelet aggregation and aPTT value were evaluated by geometric mean ratio for the AUC 3 to 30 minutes after dosing. Bleeding time was measured five minutes after administration. Data analysis showed that the ratio of platelet aggregation for aspirin combined with sugammadex to aspirin alone was 1.01. The ratio of bleeding time for sugammadex/aspirin to aspirin/placebo was 1.20. The results showed no clinically significant reduction in platelet aggregation in sugammadex 4 mg/kg administration combined with aspirin [9].

A study by Raft et al. aimed to analyze the effect of sugammadex in usual clotting and bleeding tests in a clinical environment [17]. It was a prospective observational study in which 142 patients were included. The patients, scheduled for elective laparotomy, were divided into three groups to receive sugammadex 2 mg/kg, 4 mg/kg, and placebo. aPTT, PT, hemoglobin (Hb), and hematocrit (Hct) were measured right before and after sugammadex administration. Analysis showed no correlation of sugammadex to prolonged bleeding time or lower Hb level [17].

Moon et al. compared postoperative coagulation profiles in living donors undergoing hepatectomy [18]. The two patient groups received either sugammadex 4 mg/kg or pyridostigmine 0.25 mg/.kg to reverse rocuronium-induced neuromuscular blockade. Endpoints were laboratory testing of PT, aPTT, Hb, and platelet count, as well as a clinical evaluation of postoperative bleeding. Out of 992 donors, 383 received pyridostigmine, and 609 received sugammadex as a reversal agent. There were no significant differences between the two groups in Hb, platelet count level drop, PT, or aPTT values. Two incidents of hemorrhage 24 hours postoperatively (0.3%) were reported in the pyridostigmine group, while no such incidents occurred in the sugammadex group. Anesthetic time, as well as in-hospital postoperative stay, were significantly longer in the pyridostigmine group compared to the sugammadex group.

Tas et al. investigated the effect of sugammadex in coagulation values and postoperative bleeding after septoplasty [19]. In this randomized, controlled study, 50 patients were divided into two groups to receive either neostigmine or sugammadex. Blood samples were acquired 120 minutes postoperatively, and PT, aPTT, and INR values were compared to the respective preoperative values. Postoperative bleeding was evaluated by the blood amount absorbed by the nose tampon during the first three hours after surgery. Postoperative bleeding was significantly higher in the sugammadex group. No difference in the PT, aPTT, or INR values was found between the two groups. Sugammadex was correlated with more postoperative bleeding compared to neostigmine.

de Kam et al. also conducted a two-part, randomized, double-blind, placebo-controlled, four-period crossover study in healthy males in 2014 [20]. Each subject received 40 mg of enoxaparin, 5,000 units of unfractionated heparin (UFH), or placebo followed by 4 or 16 mg/kg sugammadex or placebo. Anti-Xa activity and aPTT were evaluated for 30 minutes after the sugammadex/placebo administration. Geometric mean ratios (GMRs) and their two-sided 90% confidence limits were obtained to compare the sugammadex group versus the placebo group. They concluded that neither of the sugammadex dosages resulted in a clinically important effect, as this was specified *a priori* [20].

In the largest trial included in this review, Rahe-Mayer et al. investigated the effect of sugammadex in postoperative bleeding and clotting time [21]. It was a randomized, double-blind study, including patients undergoing total knee or hip replacement, as well as hip fracture surgery, while already on thromboprophylaxis medication. Patients received either sugammadex 4 mg/kg or standard care (neostigmine or nothing) to reverse the neuromuscular blockade induced by rocuronium or vecuronium. Out of 1,198 randomized patients, 1,184 finally received either sugammadex (n = 596) or standard care (n = 588). Hemorrhage incidents reported 24 hours postoperatively were 17 (2.9%) in the sugammadex group and 24 (4.1%) in the standard care group. A 5.5% increase in aPTT value, as well as 3.0% in the PT value, was recorded in the sugammadex group compared to the standard care group 10 minutes after the reversal agent administration. There was no difference between sugammadex and the standard care group in terms of blood loss measurement. Researchers concluded that sugammadex induced a limited, temporary increase in PT and aPTT values, but this was not correlated to augmented hemorrhage risk compared to standard care.

Targeting a specific surgical population, Carron et al. studied the effect of sugammadex in coagulation among morbidly obese patients undergoing bariatric surgery [22]. The two different groups received either sugammadex 2 mg/kg or 4 mg/kg, whereas no placebo group was assigned. There was a statistically important difference in the aPTT value before and after the sugammadex administration but not between the two different groups. As for the PT value, no statistically important difference was observed in any of the groups.

In the most recent study done in 2020, Kang et al. allocated 100 patients undergoing elective arthroscopic shoulder surgery in two groups to receive either sugammadex 2 mg/kg or sugammadex 4 mg/kg [23]. Blood samples were obtained before and 15 min after sugammadex administration. Laboratory testing included PT, aPTT, platelet count, and thromboelastography (TEG)-derived measurements. According to the results, the PT value was significantly greater after the sugammadex administration in both groups with no difference when the two groups were compared. Nevertheless, even the prolonged PT values did not exceed the normal range. There was no difference reported for aPTT values.

The characteristics of the nine studies concerning the type, the conducting time, the compared groups, and the endpoints are summarized in Table 1. 

**Table 1:** Characteristics of the Nine Studies Included in the Review *maximum value within 12 hours after administration aPTT: activated partial thromboplastin time; AUC: area under the curve; INR: international normalized ratio; PT: prothrombin time; UFH: unfractionated heparin

Study	Study type	Number of participants	Groups	Endpoints	Results concerning PT/aPTT
de Kam et al. (2014) [8]	Randomized three period crossover study	8	1) Placebo; 2) sugammadex 4 mg/kg; 3) sugammadex 16 mg/kg	AUC_2-60 min_, aPPT-*MAX_0-12 hr_, PT-*MAX_0-12 hr_, geometric mean ratio	PT and aPTT dose-dependent increase in the sugammadex groups
de Kam et al. (2013) [9]	Randomized crossover study	26	1) Sugammadex 4 mg/kg + aspirin; 2) sugammadex 4 mg/kg; 3) placebo; 4) placebo + aspirin; 5) aspirin	Platelet aggregation, aPPT, PT	PT and aPTT increase in sugammadex groups
Raft et al. (2015) [17]	Prospective observational study	142	1) Placebo; 2) sugammadex 2 mg/kg; 3) sugammadex 4 mg/kg	aPPT, PT, hemoglobin (Hb), hematocrit (Ht)	No increase in PT and/or aPTT in any of the groups
Moon et al. (2018) [18]	Retrospective observational study	992	1) Sugammadex 4 mg/kg; 2) pyridostigmine 0.25 mg/kg	PT (INR), aPPT, hemoglobin, platelet count	PT↑ PT↑; no difference between the two groups
Tas et al. (2015) [19]	Randomized control study	50	1) Sugammadex 2 mg/kg; 2) neostigmine 0.05 mg/kg + atropine 0.02 mg/kg	PT, aPPT, bleeding events	No difference in PT/ aPTT values. More bleeding in sugammadex group.
de Kam et al. (2014) [20]	Randomized crossover study	Part 1: 12 Part 2: 40	Period 1: enoxaparin 40 mg and sugammadex 4 mg/kg or sugammadex 16 mg/kg or placebo; Period 2: UFH 5000 IU and sugammadex 4 mg/kg or sugammadex 16 mg/kg, or placebo. Period 3: Placebo and sugammadex 4 mg/kg or sugammadex 16 mg/kg, or placebo; Period 4: Placebo	Anti-Xa activity and aPTT Geometric mean ratios	Dose-dependent aPTT and PT increase in sugammadex groups.
Rahe-Meyer et al. (2014) [21]	Randomized control study	1,184	1) Placebo or neostigmine; 2) sugammadex 4 mg/kg	PT (INR), aPPT	PT and aPTT value increase in sugammadex group
Carron et al. (2018) [22]	Prospective observational study	60	1) Sugammadex 2 mg/kg; 2) sugammadex 4 mg/kg	Blood coagulation, postoperative bleeding	PT -/ aPTT ↑ in both groups
Kang et al. (2020) [23]	Randomized study	100	1) Sugammadex 2 mg/kg; 2) sugammadex 4 mg/kg		PT ↑/ aPTT - in both groups

According to Table 1, three out of nine studies were randomized crossover trials [8-9, 20], two were randomized controlled trials [19, 21], one was a randomized trial without a control group [23], two were prospective observational studies [17, 22], and one was a retrospective observational study [18]. In three out of nine trials, sugammadex was used in the dosage of 4 mg/kg [9, 18, 21], in three out of nine trials the sugammadex dosage was 2 mg/kg and 4 mg/kg [17, 22-23], in two out of nine trials, sugammadex 4 mg/kg and 16 mg/kg were used [8, 20], and finally, in one out of nine, the dosage for sugammadex was 2 mg/kg [19].

Moreover, in two out of nine trials, the control group received a placebo [8], [17]. In one out of nine, the control groups received placebo, aspirin and placebo with aspirin [9]. In one trial, the control groups received a placebo, a placebo with enoxaparin, or a placebo with heparin [20]. In one out of nine, the control group received pyridostigmine [18]. In two out of nine trials, the control group received neostigmine [19] or either neostigmine or a placebo [21], whereas two trials did not include a control group [22-23].

PT and/or aPTT were found to increase after sugammadex administration in seven out of the nine studies [8-9, 18, 20-23]. However, the increase seemed to be transient and dose-dependent. Indeed, the greatest increase occurred in the sugammadex 16 mg/kg dosing, which, in a clinical setting, is used for immediate neuromuscular blockade reversal. In such cases, the surgery is most probably postponed so a bleeding predisposition is no longer a concern. 

The authors intended to conduct a meta-analysis using the data of the studies included in this review. However, this was not possible as the effect size measures in these studies were not comparable and the domain for which the effect would be meta-analyzed was not clearly delimited.

## Conclusions

According to the literature, sugammadex temporarily increases aPTT and PT values, as compared to traditional reversal agents. Nevertheless, there is heterogeneity between the trials’ findings. Therefore, a meta-analysis of the provided data could not be conducted. Although the transient changes of the examined parameters do not seem to have a clinical impact, sugammadex’s interference with coagulation has been said to be just an *in vitro* artifact. More well-designed studies are needed to make prudent conclusions.
